# Dr. Frederick Llewellyn Hovey Lewis

**Published:** 1914-06

**Authors:** 


### OBITUARY

---

Readers are requested to report promptly the death of all physicians in Western New York, or former residents of this region, or graduates of any medical school in Western New York, and to notify the families of the deceased of our desire to publish adequate obituary notices.

---

Dr. Frederick Llewellyn Hovey Lewis, New York Homoeopathic Medical College, 1865, died at his home in Rochester, April 12, aged 85. He was a Unitarian clergyman and was one of the founders of a colony in Massachusetts to carry out ideals of social life. He was said to have been the original of Laurie in "Little Women."

